# Fellow-Workers and the Roll of Honour

**Published:** 1915-01-02

**Authors:** 


### ROUND THE WORLD.

[The Editor Invites Early Information and Contributions Marked "Fellow-Workers."]

### Fellow-Workers and the Roll of Honour.

Mr. William Tindall Lister, who has just been appointed consulting ophthalmic surgeon to the British Forces Oversea, is ophthalmic surgeon to the London Hospital. Originally a student of Cambridge and University College Hospital, Mr. Lister became an F.R.C.S. Eng. in 1895, and, turning his attention to ophthalmology, became assistant surgeon to the Royal London Ophthalmic Hospital (Moorfields), ophthalmic surgeon to the Children's Hospital at Great Ormond Street, and assistant ophthalmic surgeon to the Great Central Ophthalmic Hospital—important posts which he has since relinquished. He is the author of numerous writings on his special subject.

Sir Charles Bent Ball, who is now one of the consulting surgeons to the troops in Ireland, is the well-known Regius Professor of Surgery in Dublin University and honorary surgeon to the King in Ireland. Educated at Trinity College, Dublin, Sir Charles, who now ranks as a lieutenant-colonel, had a brilliant academic career, in the course of which he won various important prizes and a gold medal in natural science. Becoming an M.D., M.Ch., F.R.C.S.I., his services in the advancement of modern surgery gained him the rarely awarded distinction of honorary F.R.C.S. Eng. Lieutenant-Colonel Sir Charles Ball has for many years been attached to the Sir Patrick Duns Hospital, and has also served for some time on the General Medical Council. In 1902 he went to San Francisco to deliver the Lane lectures, whilst in the following year he was the Erasmus Wilson lecturer at the Royal College of Surgeons. A Lord Chancellor's consulting visitor in lunacy, a medical referee under the Workmen's Compensation Act, and formerly Commissioner for Education in Ireland, he was knighted in the year of his American visit, and created a baronet three years ago.

Sir Thomas Myles, M.D., F.R.C.S.I., who has also been appointed consulting surgeon to the troops in Ireland, with the rank of lieutenant-colonel, has been surgeon to the Richmond Hospital in Dublin for some twenty-five years, and is a past president of the Irish College of Surgeons. A honorary surgeon to the King in Ireland, and an ex-member of the General Medical Council, Lieutenant-Colonel Sir Thomas Myles was knighted in 1902. He was formerly secretary to the Dublin Hospitals Commission, and examined for the Conjoint Board of the Royal Colleges for some years.

Mr. James Thomas Jackman Morrison, M.Sc. Birm., M.B., B.C. Cantab., F.R.C.S. Eng., the senior surgeon to the Queen's Hospital, Birmingham, should by now have arrived in Serbia with the large ambulance party of which he is in charge. Mr. Morrison, who is an old Guy's man, particularly distinguished himself as a student, amongst other awards gaining the gold medal in surgery at his hospital. After qualification, he held the posts of house surgeon at Guy's and senior demonstrator in anatomy at Cambridge, and ultimately became attached to the staff of the Queen's Hospital, which he has served with distinction for many years. Apart from his work there, he occupies the chair of forensic medicine in Birmingham University, where he also examines in clinical surgery. Having served in the Territorial branch of the R.A.M.C. for some little time, this well-known Midland consultant held the rank of major therein when the war broke out, and the ambulance party which he has taken out to the Near East includes thirty-one doctors and twenty-seven nurses.

Dr. Ferdinand Campion Batchelor, the veteran professor of surgery at Otago University, who has come over as lieutenant-colonel with the New Zealand Expeditionary Force, in spite of his threescore years and ten, is also an old Guy's man. Qualifying in 1871, Lieutenant-Colonel Batchelor eventually settled in New Zealand, and became attached to the Dunedin Hospital. Some years ago he gave an account of his experiences of abdominal surgery in the *Australian Medical Journal*. It is interesting to note that one of his sons, Mr. Ferdinand Stanley Batchelor, F.R.C.S. Eng., followed him at Guy's, and subsequently in practice at Dunedin.

Dr. Herbert George Graham Cook, who has taken charge of the "Glamorgan and Monmouthshire Hospital for French Wounded," is the senior assistant surgeon to the King Edward VII. Hospital at Cardiff, and medical officer to the prison there. A student of St. Bartholomew's, he was awarded honours in medicine and forensic medicine at the M.B. Lond. in 1890, while in the following year he became an F.R.C.S. Eng., and won the gold medal at the B.S. Lond. Aspiring to still further distinctions, Dr. Cook took the D.P.H. Cambridge in 1893, and in the same year carried off the gold medal at the M.D. Lond. Before settling in Cardiff he was house physician at "Bart.'s," and house surgeon to the Victoria Hospital for Children. Dr. Cook's hospital which is to be maintained on the Continent will accommodate a hundred patients, and the party which has gone out with him includes eight nurses, two dressers, and ten orderlies. It is understood that a keen supporter of this scheme has provided a complete *x*-ray outfit, while other friends have subscribed sufficient funds to run the institution for several months.

Mr. Frederick William Samuel Davies, M.R.C.S., L.R.C.P., who will be one of the medical officers under Dr. Herbert Cook, is senior anæsthetist to the King Edward VII. Hospital at Cardiff, and physician to the French Consulate there; while Mr. Stuart Cornwallis Cresswell, M.R.C.S., L.R.C.P., his colleague, is surgeon to the Merthyr General Hospital, medical officer and public vaccinator to the Merthyr Tydvil Union, and resides at Dowlais.

Dr. John Freeman, who has journeyed to Galicia in search of a special variety of cholera bacillus, is assistant bacteriologist at St. Mary's Hospital, and assistant in the famous inoculation department there controlled by Sir Almroth Wright. Before proceeding to St. Mary's for clinical work Dr. Freeman was at Oxford, and qualified M.B., B.Ch. Oxon. in 1905, subsequently taking the M.D. degree of his University. After qualification he devoted himself to bacteriology, and has done important work in connection with inoculation therapy, particularly in regard to immunisation against hay fever.

